# Analyses of basal media and serum for in vitro expansion of suspension peripheral blood mononucleated stem cell

**DOI:** 10.1007/s10616-014-9819-8

**Published:** 2015-08-01

**Authors:** Shahrul Hisham Zainal Ariffin, Nur Akmal Mohamed Rozali, Rohaya Megat Abdul Wahab, Sahidan Senafi, Intan Zarina Zainol Abidin, Zaidah Zainal Ariffin

**Affiliations:** 1 School of Biosciences and Biotechnology, Faculty of Science and Technology, Universiti Kebangsaan Malaysia, 43600 Bangi, Selangor Malaysia; 2 Department of Orthodontic, Faculty of Dentistry, Universiti Kebangsaan Malaysia, Jalan Raja Muda Abdul Aziz, 50300 Kuala Lumpur, Malaysia; 3 Department of Microbiology, Faculty of Applied Sciences, MARA University of Technology, 40450 Shah Alam, Selangor Malaysia

**Keywords:** Basal media, Cell differentiation, Cell expansion, Peripheral blood, Serum, Suspension stem cell

## Abstract

Transplantation of stem cells requires a huge amount of cells, deeming the expansion of the cells in vitro necessary. The aim of this study is to define the optimal combination of basal medium and serum for the expansion of suspension peripheral blood mononucleated stem cells (PBMNSCs) without resulting in loss in the differentiation potential. Mononucleated cells were isolated from both mice and human peripheral blood samples through gradient centrifugation and expanded in α-MEM, RPMI, MEM or DMEM supplemented with either NBCS or FBS. The suspension cells were then differentiated to osteoblast. Our data suggested that α-MEM supplemented with 10 % (v/v) NBCS gives the highest fold increase after 14 days of culture for both mice and human PBMNSCs, which were ~1.51 and ~2.01 times, respectively. The suspension PBMNSCs in the respective medium were also able to maintain osteoblast differentiation potential as supported by the significant increase in ALP specific activity. The cells are also viable during the differentiated states when using this media. All these data strongly suggested that α-MEM supplemented with 10 % NBCS is the best media for the expansion of both mouse and human suspension PBMNSCs.

## Introduction

Hematopoietic stem cells (HSCs) are adult stem cell that can differentiate into myeloid and lymphoid progenitors (Doulatov et al. 2010). There are several sources of HSCs, namely cord blood (Broxmeyer et al. 2011; Ferreira et al. 2012), bone marrow (Fukata et al. 2013; Schirhagl et al. 2011) and peripheral blood (Muhammad Dain et al. 2011; Ruzanna et al. 2012; Shahrul Hisham et al. 2008). It is relatively easier for the donor to donate peripheral blood as opposed to bone marrow as the means of harvesting bone marrow is typically painful and results in extended recovery time for the donor (Korbling 2001). Peripheral blood is also shown to be a good source of stem cells for transplantation by having significantly lower prevalence of molecular and cytogenetic relapse and a better disease-free survival rate as opposed to bone marrow stem cell transplantation (Korbling 2001).

Currently, HSCs remain as the best characterized adult stem cells, with the studies stretching back to the 1960s (Armstrong et al. 2012; Lacadie and Zon 2011). HSCs are shown to be able to differentiate into osteoclasts (Muhammad Dain et al. 2011; Ruzanna et al. 2012; Shahrul Hisham et al. 2008), osteoblasts (Hofmann et al. 2013; Muhammad Dain et al. 2011; Ruzanna et al. 2012; Shahrul Hisham et al. 2008), dendritic cells (Hamdorf et al. 2011), cardiomyocytes (Fukata et al. 2013), chondrocytes (Ogawa et al. 2010; Shahrul Hisham et al. 2008), and fibroblasts and adipocytes (Ogawa et al. 2010). It has previously proven that stem cells of both mesenchymal (adherent) and hematopoietic (suspension) origin are present in the population of peripheral blood mononucleated cells (Muhammad Dain et al. 2011; Ruzanna et al. 2012; Shahrul Hisham et al. 2008).

Since PBMNSCs have a potential to differentiate into multiple cells, clinical trials on hematopoietic stem cells have been done to evaluate its possible application in the treatment of multiple sclerosis, rheumatoid and juvenile idiopathic arthritis and genetic blood diseases such as sickle cells (Trounson et al. 2011). These transplants require a high amount of cells, where up to 10^8^ cells per kg of body weight have been used per transplant (Tse and Laughlin 2005). Taking account of the low amount of PBMNSC that can be isolated per mL of peripheral blood, in vitro expansion of the cells is absolutely necessary prior to in vivo transplantation of the cells. Since the cells are cultured in an environment outside of their niche, optimization of the culture conditions, especially the basal medium and serum are important to define the best medium concoction for ensuring a high proliferation rate of PBMNSC while still leaving its multipotential properties intact.

Currently, stem cell culture has been leaning towards serum-free media as the presence of xenogeneic compounds inside the bovine derived serum is claimed to cause a negative immune response when used for transplantation purposes (Mannello and Tonti 2007). However, serum-free media would require the addition of growth factors (Mannello and Tonti 2007; Tonti and Mannello 2008), adding cost to the culture system. Moreover, as research advances, any internal antigens resulting from the usage of animal sera can be removed prior to transplantation, allowing the cells to be expanded in bovine-sourced serum with no immunogenic effect to the recipient (Spees et al. 2004). This would definitely reduce the cost of stem cell culture considerably. In regards to suspension PBMNSCs, lack of research could be the reason behind insufficient data on the optimum culture medium. Some media combinations that have been used include RPMI1640 + 10 % fetal bovine serum (FBS) + 0.1 % penicillin–streptomycin (Potdar and Subedi 2011), α-MEM + 20 % FBS + 2 mM l-glutamine + 55 μM 2-mercaptoethanol + 100 U/ml penicillin + 100 μg/ml streptomycin (Akiyama et al. 2012) and α-MEM + 10 % newborn calf serum (NBCS) + 1 % penicillin–streptomycin (Muhammad Dain et al. 2011; Ruzanna et al. 2012; Shahrul Hisham et al. 2008). Thus, the focus of this study is to determine the best combinations of basal media and serum for the optimal proliferation of the stem cells.

## Material and method

### Isolation of peripheral blood mononucleated cells

Blood was obtained from 6 to 8 weeks old ICR strain mice (Animal House, Universiti Kebangsaan Malaysia) through cardiac puncture. Human blood samples were drawn via vein puncture from subjects of 20–25 years old with informed consent. The procedures were done as approved by our institutional ethical committee (UKM 1.5.3.5/244/02-01-02-SF1052). Isolation of peripheral blood mononucleated cells were conducted as previously described with minor modifications (Ruzanna et al. 2012; Shahrul Hisham et al. 2008). Briefly, mice blood samples were diluted one times with 1X phosphate buffered saline; PBS (Sigma, St. Louis, MO, USA) and human blood samples were diluted three times with Hanks’ balanced salt solution; HBSS (Sigma). The diluted blood was layered on Ficoll-paque™ PLUS (GE Healthcare, Uppsala, Sweden) at a ratio of undiluted blood to Ficoll 1:1.5, followed by centrifugation at 400*g* for 30 min at room temperature. The mononucleated cells were then harvested and washed three times with PBS. After the final wash, the cells were resuspended in PBS and the cell viability analyzed through trypan blue cell exclusion assay.

### Proliferation of peripheral blood mononucleated cells

Four types of basal media were used in this study—α-MEM (α-Minimal Essential Medium, Biowest, Kansas City, MO, USA, Cat. No. P0440), DMEM (Dulbecco’s Modified Eagle’s Medium, Gibco, Grand Island, NY, USA, Cat. No. 12800-017), MEM (Minimal Essential Medium, Biowest, Cat. No. P0451) and RPMI-1640 (Roswell Park Memorial Institute Medium 1640, Gibco, Cat. No. 31800-022) and two types of serum, namely FBS (fetal bovine serum, Gibco) and heat-inactivated NBCS (newborn calf serum, Gibco). The proliferation medium was made up by basal medium, 10 % (v/v) serum and 1 % (v/v) penicillin–streptomycin (Invitrogen, Carlsbad, CA, USA). For proliferation studies, freshly isolated cells were seeded in 24-well plate at a density of 1 × 10^5^ cells/mL in proliferation medium and counted every day for a total of 14 days. The cells were sub-cultured and re-seeded at the original seeding number once the number of cells exceeded 1 × 10^5^ cells/mL.

### Differentiation potential analysis

After 14 days of expansion in proliferation medium, the suspension mononucleated cells were subjected to osteoblast differentiation. All chemicals were supplied by Sigma, unless stated otherwise. The cells were seeded in 96-well plates at a density of 1 × 10^5^ cells/mL in 200 µL of proliferation medium supplemented with 50 µg/mL ascorbic acid and 10 mM β-glycerophosphate and cultured for an additional 14 days. Cell viability and ALP activities were analyzed during the differentiation process. For ALP analysis, the cells were incubated at 37 °C in 2 mM MgSO_4_, 6 mM pNPP (*p*-nitrophenyl phosphate), 0.1 % (v/v) triton X-100 and 0.1 M bicarbonate-carbonate buffer, pH 10. After 30 min, the reaction was stopped by the addition of 1 M NaOH and the absorbance was read at 405 nm using a Model 680 Microplate Reader (BioRad, Hercules, CA, USA). The ALP specific activity can be described as the activity of ALP enzyme per mg of total protein. The total protein content was determined using Bradford assay. Bradford solution was prepared from Coomasie Blue G250 (Sigma) according to protocol by Kruger (2009).

### Statistical analysis

Paired *t* test was calculated using statistical software MINITAB^®^ v14 and *p* < 0.05 was accepted to be statistically significant.

### Reverse transcriptase polymerase chain reaction

Total RNA was extracted from suspension cells using TRI Reagent (Sigma) and one-step RT-PCR was conducted using Access RT-PCR System Kit (Promega, Madison, WI, USA) in Mastercycler Gradient PCR machine (Eppendorf, Hauppauge, NY, USA). Approximately 1 µg of RNA template was subjected to first strand cDNA synthesis at 45 °C for 45 min, inactivation of the reverse transcriptase at 94 °C for 2 min and PCR amplification which comprised of 40 cycles of denaturation at 94 °C for 30 s, annealing at 54– 63 °C for 1 min and extension at 68 °C for 2 min. The reaction was terminated with a final elongation step at 68 °C for 7 min. The primer sequences used, annealing temperatures and product size are listed in Table 1. GAPDH was used as internal control for the RT-PCR reaction.

**Table 1:** Primer sequences, product sizes and the annealing temperatures used in the reverse transcriptase PCR

Sample	Primer	Sequence (5′–3′)	Annealing temperature (°C)	Product size (bp)
Mice	GAPDH	CAACGGCACAGTCAAGG	62	717
		AAGGTGGAAGAGTGGGAGT		
	CD38	ACGCTGCCTCATCTACACTC	56	343
		TTCACTCCAATGTGGGCAAG		
	SCA-1	GGCAGCAGTTATTGTGGATT	63	167
		CAGTTCCCAATGGAGGCA		
	CD105	AGGCTGAAGACACTGACGACCAT	63	356
		CTTGGCTGTTCGGCTCTGGATG		
	CD73	AGGGAGTGGGTAAGG	57	765
		GGAGTCGCACAGGAG		
Human	GAPDH	CCATGGAGAAGGCTGG	55	195
		CAAAGTTGTCAGGATGACC		
	SLAMF1	CTCTGCGTTCTGCTCCTAC	54	403
		TTGGTCACTTCTGGGTCTG		
	CD133	CCAAGGACAAGGCGTTCA	57	264
		GCACCAAGCACAGAGGG		
	CD105	GCTCCCTCTGGCTGTTG	61	290
		TTACACTGAGGACCAGAAGC		
	CD90	TGGACCAGAGCCTTCG	54	143
		TCGGGAGCGGTATGTG		

## Result and discussion

### Effect of different basal media and serum combinations on the proliferation of mice and human peripheral blood mononucleated stem cells

Different types of cells would require different growth requirements, giving out the need to optimize the media to ensure the expanded cells are of both quantity and quality. Some of the variables that have been manipulated for this purpose include cytokines cocktails (Andrade et al. 2010; Sotiropoulou et al. 2006; Yao et al. 2004; Zhang and Lodish 2005), serum (Azouna et al. 2012; Carrancio et al. 2008; Eslaminejad et al. 2009; Shahdadfar et al. 2005), basal medium (Chen et al. 2010; Sotiropoulou et al. 2006), method of medium change (Choi et al. 2010) and culture environments (Chen et al. 2010; Saha et al. 2011; Sotiropoulou et al. 2006). The previous work done in order to find optimal media for stem cells showed that some cells thrive better in one medium and vice versa. The optimal basal media and serum has not been studied before for suspension peripheral blood mononucleated stem cells (PBMNSC). Since suspension PBMNSC is a potential source of stem cells, it is important to develop an optimal culture system for the cell.

Stem cells of mesenchymal and hematopoietic origin have previously been confirmed to be present in population of peripheral blood mononucleated cells (Ruzanna et al. 2012; Shahrul Hisham et al. 2008). In order to determine the optimal combination of basal medium and serum for the expansion of suspension PBMNSC, the proliferation analysis of the suspension PBMNSC in different proliferation media was carried out for 14 days. In this study, each one of the basal media was supplemented with either fetal bovine serum (FBS) or newborn calf serum (NBCS) at concentration of 10 %. This is because studies involving stem cells have used from 10 up to 20 % of serum concentration in the culture medium depending on the cell types (Akiyama et al. 2012; Broxmeyer et al. 2011; Carrancio et al. 2008; Chen et al. 2010; Lindroos et al. 2010; Lysdahl et al. 2013; Saha et al. 2011; Shahdadfar et al. 2005; Sotiropoulou et al. 2006). This study focused on the most frequent serum concentration used for stem cell culture, which is 10 %. Further study would be required in order to determine the optimal serum concentration for the culture of suspension PBMNSC.

**Fig. 1:**
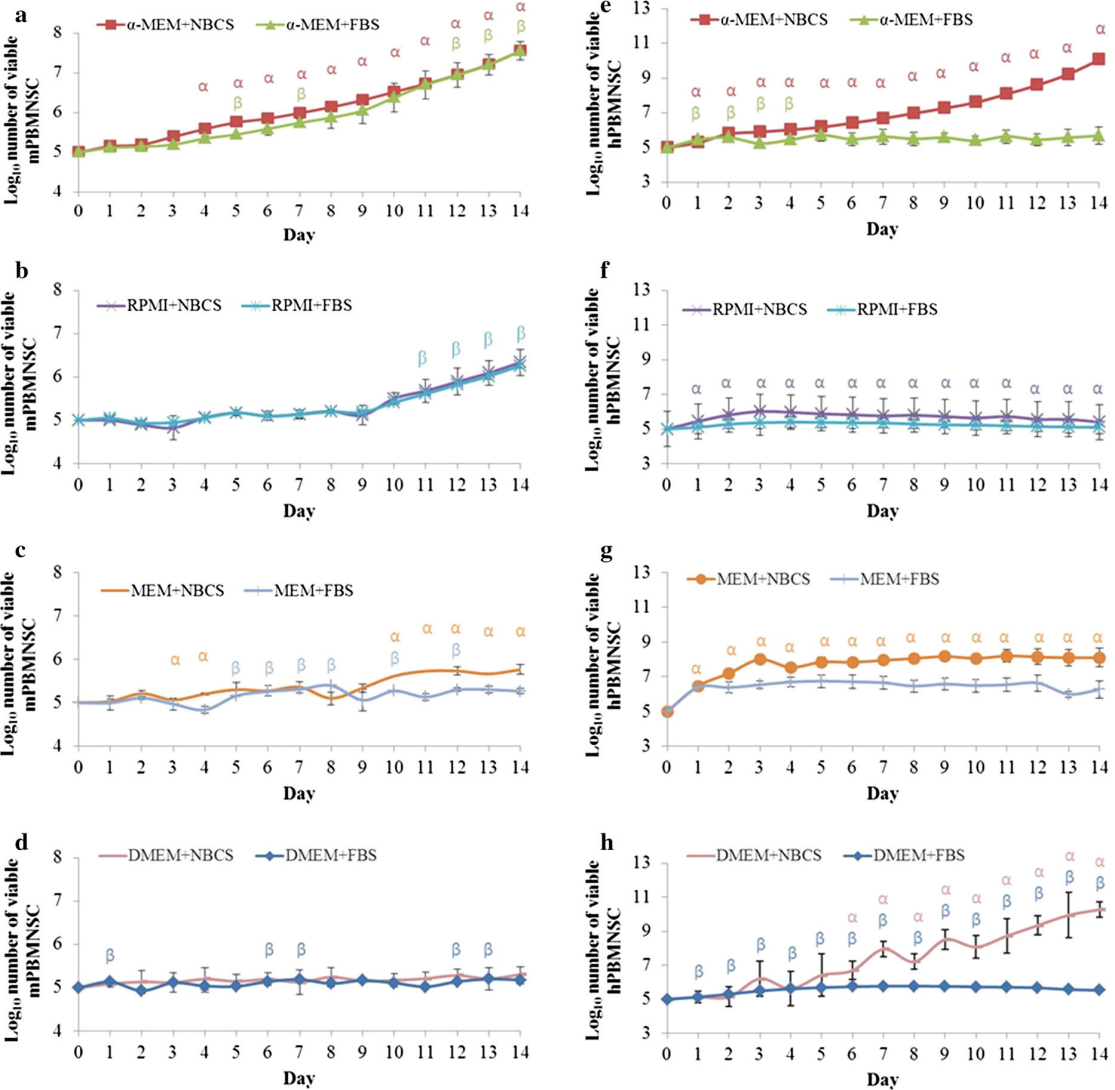
Proliferation of mice (**a**–**d**) and human (**e**–**h**) peripheral blood mononucleated stem cells (PBMNSC) for 14 days. Freshly isolated cells were seeded at a density of 1 × 10^5^ cells/mL and the amount of viable cells was counted using trypan blue cell exclusion assay. Cells were re-seeded at the original seeding number when the number of cells exceeded 1 × 10^5^ cells/mL. Values were expressed in mean log number of cells ± standard error (n = 3). The point marked as α is significant (*p* < 0.05) relative to number of cells at day 0 for cells cultured in medium supplemented with newborn calf serum (NBCS) and β for the cells cultured in fetal bovine serum (FBS)

**Fig. 2:**
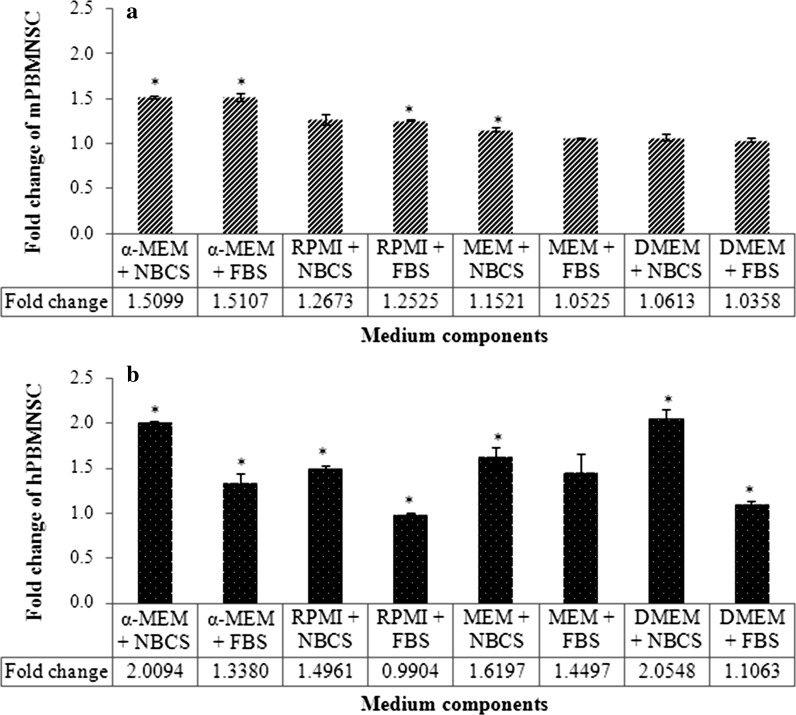
Fold change of the number of mouse (**a**) and human (**b**) peripheral blood mononucleated stem cells (PBMNSC) cultured in different combinations of basal media and serum after being cultured for a total of 14 days. The values are represented by the mean fold change ± standard error (n = 3). Cells showing a significant increment as compared to day 0 *p* < 0.05) and fold change of more than ~2 were subjected to differentiation analysis to osteoblast to analyze whether the cells retained their differentiation potential after being expanded inside their respective media

The result obtained showed that both human and mouse suspension PBMNSC require different growth conditions. While the former was able to expand in DMEM, mouse suspension PBMNSC showed only a very small to no significant increase when cultured in the same medium (Fig. 1d, h). The fold change for the cells cultured in different combinations of basal media and serum after 14 days was then calculated. Surprisingly, for mouse suspension PBMNSC, the fold change for cells cultured in α-MEM + NBCS and α-MEM + FBS were both significant (*p* < 0.05) and almost the same (Fig. 2).

However, the proliferation of mouse suspension PBMNSC in α-MEM + NBCS had increased significantly (*p* < 0.05) starting from day 4, unlike α-MEM + FBS, where the significant increase in cell number during proliferation can only be seen starting from day 12, signifying a longer period of lag phase (Fig. 1a, b). For the rest of the media, despite the fact that they were able to support and maintain the cells in vitro, they were not good media for the expansion of mouse suspension PBMNSC (Fig. 1b, c, f, g). The increase of mouse suspension PBMNSC after 14 days in culture was less than ~twofold for RPMI + FBS and MEM + NBCS while the rest of the media did not show any statistically significant (*p* < 0.05) changes in the cell number compared to day 0 (Fig. 2).

For human suspension PBMNSC, out of all eight media tested, the cells cultured in all basal medium supplemented with NBCS were observed to be able to support the suspension PBMNSC proliferation. Human suspension PBMNSC cultured in α-MEM + NBCS and DMEM + NBCS showed the highest significant increase after 14 days, followed by RPMI + NBCS and MEM + NBCS. However, all cells cultured in FBS supplemented medium showed either a very low increment of less than ~2 times (α-MEM + FBS and DMEM + FBS), no significant change in the cell number (MEM + FBS) or even a significant decrement in the cell number, as seen in RPMI + FBS (*p* < 0.05) (Fig. 2).

The results obtained from this study were in concordance with the previous findings which showed that the effect of basal medium on cell growth varies depending on the origin and the cell type (Carrancio et al. 2008; Chen et al. 2010; Eslaminejad et al. 2009; Peister et al. 2004; Sotiropoulou et al. 2006). A study on human MSC and mouse MSC showed that Iscove’s Modified Dulbecco’s Medium (IMDM) was able to support the proliferation of mouse MSC but not human MSC (Peister et al. 2004; Sotiropoulou et al. 2006). On the other hand, for induced pluripotent stem cells, out of α-MEM, DMEM, RPMI and medium 199 (M199), only DMEM was able to support the cell growth (Chen et al. 2010). Serum types and concentrations are two variables which also require consideration in stem cell expansion. Bone marrow mesenchymal stem cells (MSC) grown in DMEM-low glucose supplemented with platelet lysate was shown to have a shorter expansion time as opposed to fetal bovine serum (FBS) supplemented medium (Carrancio et al. 2008) and equine bone marrow MSC gave the highest fold increase in 15 % FBS than 5, 10 and 20 % (Eslaminejad et al. 2009).

**Fig. 3:**
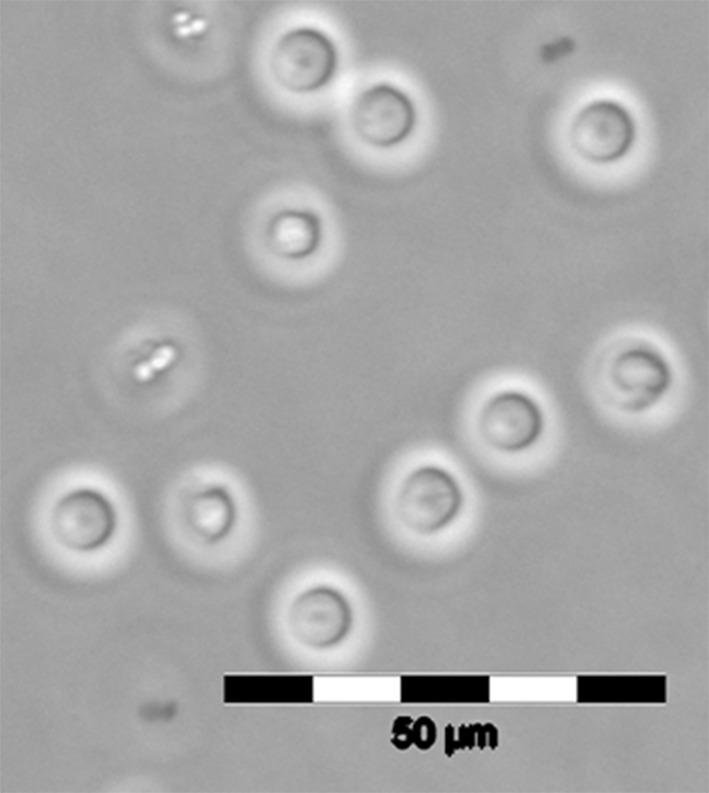
Representative image of suspension peripheral blood mononucleated stem cell in 14-day culture

The 14-day old suspension PBMNSCs were then subjected to differentiation to osteoblast to observe whether the cells were able to retain their differentiation potential after being expanded inside their respective medium. All cells showed a rounded morphology normally seen in suspension cultures irrespective of the culture medium used (Fig. 3). This signified that the different culture media used in this study did not directly affect the cells to give abnormal morphologies. Hence, we hypothesized that although the cells were able to proliferate continuously in the media and maintain their rounded, suspension form, they could lose their potential to differentiate into other cells and vice versa. This was supported by an independent experiment performed by Shahdadfar et al. (2005) which showed different type of genes would be expressed by the cells growing in different environments, and this can include the genes controlling the cell cycle and differentiation. For differentiation analysis, only the cells cultured in the media that have been successfully expanded at least 2 times of its original number, namely α-MEM + NBCS and α-MEM + FBS for mouse suspension PBMNSC, and α-MEM + NBCS, RPMI + NBCS, MEM + NBCS and DMEM + NBCS for human suspension PBMNSC are chosen. The rest of the media were not selected for the subsequent analysis due to the low numbers of suspension PBMNSC acquired after 14 days of culture. This particular method of eliminating the media which failed to support the expansion of the cells prior to other further tests was also done by Sotiropoulou et al. (2006). The cells were cultured in complete medium for 14 days prior to differentiation to osteoblast to allow depletion of the progenitor cells. This was based on molecular characterization of the suspension PBMNSC which showed that osteoblast progenitors are absent in 14 days old culture (Muhammad Dain et al. 2011; Ruzanna et al. 2012).

### Determination of osteoblastic differentiation of stem cell using ALP assay

The suspension PBMNSCs were differentiated to osteoblast and alkaline phosphatase (ALP) assay was conducted to analyze the progress of differentiation. ALP is a hydrolyzing enzyme involved in the breakdown of pyrophosphate, providing the necessary inorganic phosphate for mineralization (Orimo 2010). This enzyme has been used in a lot of studies as a marker for osteoblastic differentiation of stem cells including rabbit marrow mesenchymal stem cells (Hu et al. 2013), human marrow mesenchymal stem cells (Lysdahl et al. 2013; Prins et al. 2014; Sheehy et al. 2012) and murine adipose stem cells (Dahl et al. 2013; Jang et al. 2011).

**Fig. 4:**
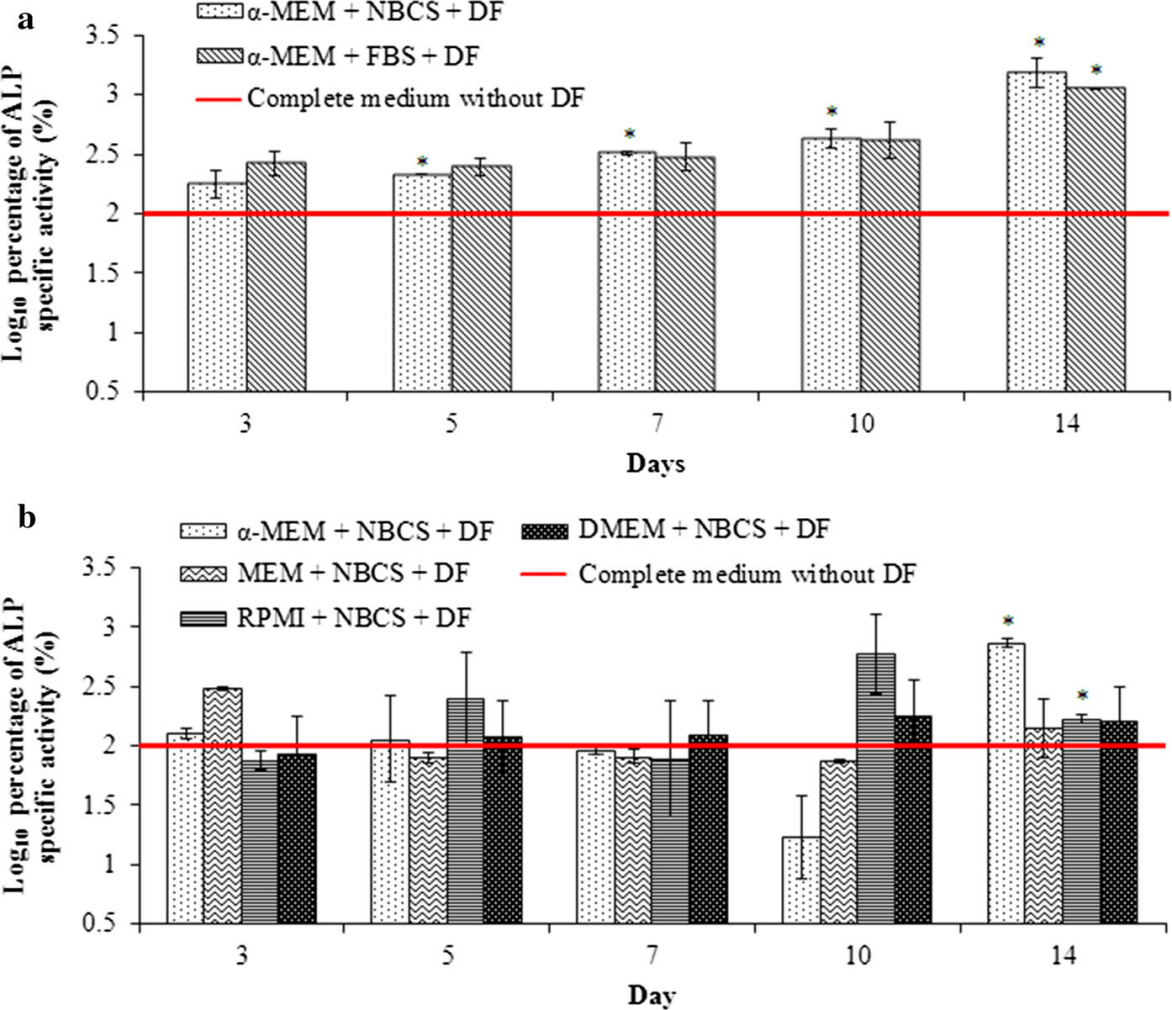
ALP specific activity of mouse (**a**) and human (**b**) peripheral blood mononucleated stem cells cultured in different combinations of basal medium supplemented with serum and differentiation factors (DF) consisting of ascorbic acid and β-glycerophosphate. The ALP activity of the cells in differentiation medium was normalized to the negative control which was cells cultured in medium without the DF. The values are expressed in terms of mean activity ± standard error (n = 3). [*significant (*p* < 0.05) as compared to negative control]

ALP specific activity was measured for the cells cultured in their respective media with differentiation factors (DF) added. Cells cultured in media without differentiation factors were used as the negative control and the reference for the normalization of the ALP specific activity. For mouse suspension PBMNSC, the ALP specific activity for cells cultured in α-MEM + NBCS + DF increased, becoming significantly different (*p* < 0.05) compared to the control starting from day 5, as opposed to cells in α-MEM + FBS + DF, whose ALP activity increased significantly only on day 14 (*p* < 0.05) (Fig. 4a). On day 14, the fold increase for the ALP specific activity for differentiated mouse suspension PBMNSC in α-MEM + NBCS and for α-MEM + FBS was 1.59 ± 0.108 and 1.53 ± 0.002, respectively. As the same basal medium has been used in this case, this result basically shows that serum choice does have an impact on the differentiation rate of the stem cells to osteoblasts. A similar observation has been seen in human mesenchymal stem cells (Shahdadfar et al. 2005) and adipose stem cells (Lindroos et al. 2010).

In case of human suspension PBMNSC, the increase in ALP specific activity became significant only on day 14 for both α-MEM + NBCS + DF and RPMI + NBCS + DF (Fig. 4b). No significant changes in the ALP level was seen in cells cultured in MEM + NBCS + DF and DMEM + NBCS + DF throughout the 14 days of culture compared to the control media. The fold increase at day 14 compared to day 0 was calculated for the media, and α-MEM + NBCS showed the highest increase (1.44 ± 0.033), than the cells in RPMI + NBCS + DF (1.11 ± 0.029). Hence, we concluded that the basal medium α-MEM provided the best environment for differentiation of human suspension PBMNSC to osteoblast as opposed to other basal media tested.

Based on all these results, it was determined that α-MEM + NBCS was the most optimal medium for the proliferation of mouse and human suspension PBMNSC whilst maintaining the stem cell ability to differentiate to other cell types. The formulations making up each basal media was compared, and one of the components separating α-MEM from the rest of the basal media used is vitamin C. Vitamin C has the ability to reduce any oxidative damage acquired by the mature cells due to its strong anti-oxidant activity (Parrinello et al. 2003; Sauer and Wartenberg 2011), downregulate cell senescence control genes such as p53 and p21 while still keeping the DNA repair machinery intact (Esteban et al. 2010; Lin et al. 2013), and maintaining the self-renewal and pluripotency by upregulating the expression of pluripotent markers such as Esrrb, Klf4, Tcl1, Eras, and Nanog (Gao et al. 2013; Wei et al. 2014). These might explain how the suspension PBMNSC self-renewal ability and its differentiation potential can be retained in vitro.

### Cell viability during osteoblastic differentiation

**Fig. 5:**
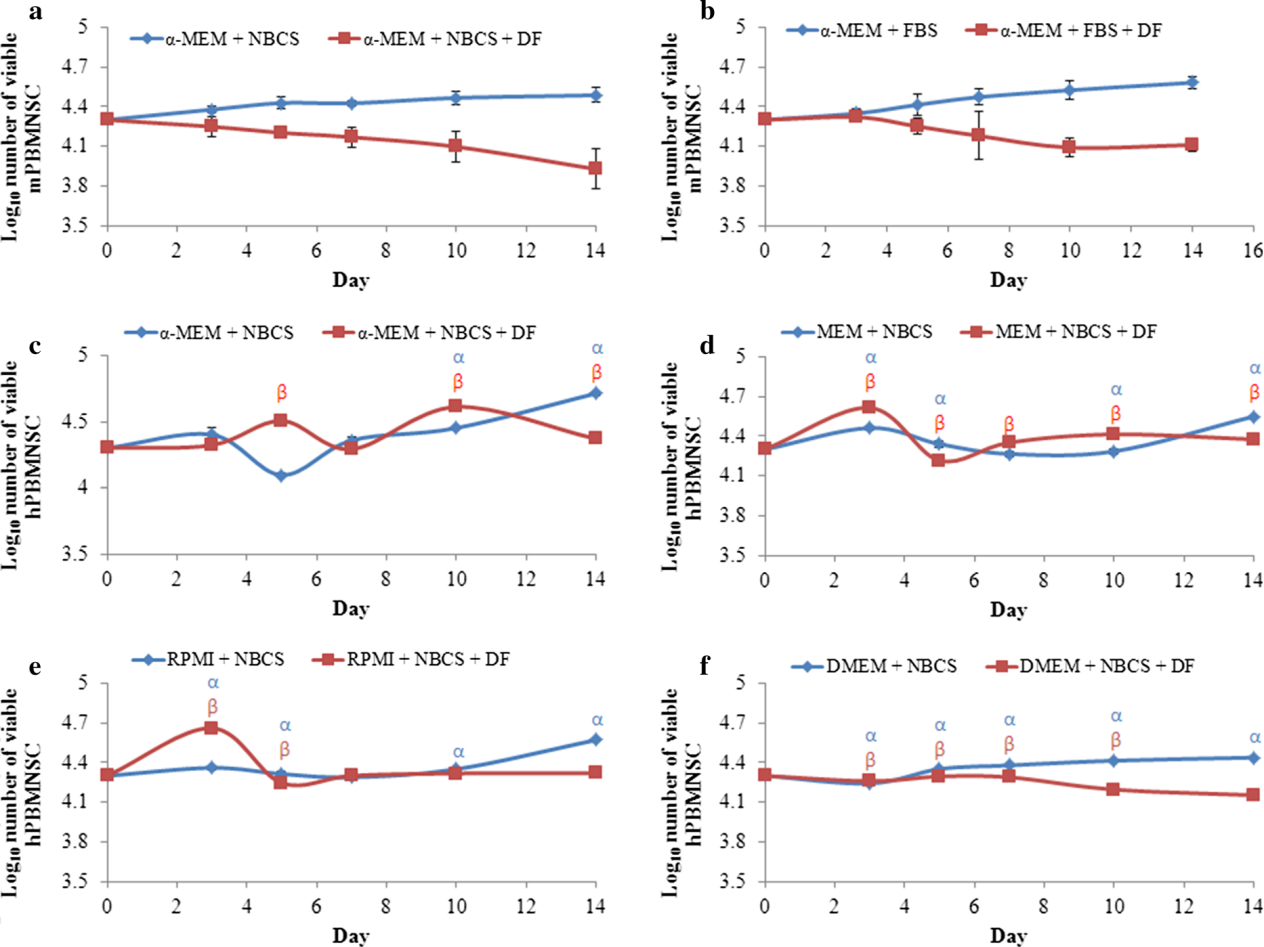
Viability analysis of mouse (**a**–**b**) and human (**c**–**f**) peripheral blood mononucleated cells cultured as static culture in differentiating medium with proliferating medium acting as a control (mean ± standard error, n = 3). Cells were cultured at a density of 1 × 10^5^ cells/mL in 200 µL per well of 96-well plate and were counted on days 3, 5, 7, 10 and 14 using trypan blue cell exclusion assay. The number of viable cells was compared with the cells on day 0. Any significant changes (*p* < 0.05) in the number of viable cells are marked with *α* for the cells cultured in newborn calf serum (NBCS) and *β* for the cells cultured in fetal born calf serum (FBS)

The viability of the cells during differentiation process was then analyzed (Fig. 5). Although mouse suspension PBMNSC were able to differentiate into osteoblast in α-MEM + NBCS by day 14 as discussed earlier, the cell viability remained stationary with no significant changes between the original cell seeding number of 1 × 10^5^ cells/mL and cell number at day 14 of differentiation (Fig. 5a). The same result was also seen for the cells in α-MEM + FBS (Fig. 5b). In case of human suspension PBMNSC, there was a significant increase that can be observed in the number of viable cells cultured in all four media tested for the differentiation study without any addition of DF (Fig. 5c-f). However, when the DF was supplemented to the media, only the cells in α-MEM + NBCS and MEM + NBCS were still able to proliferate, resulting in a significant increase in the number of viable cells, although the proliferation occurred at a slower rate than in the control medium (medium without DF) (Fig. 5c, d).

The cessation of suspension PBMNSC proliferation when induced to osteoblast can be explained by the osteoblast phenotype development process. Proliferation of osteoblastic cells depends upon the formation of bone mineralized matrix (Coelho and Fernandes 2000). When PBMNSC goes through the first stage in osteoblast development to form pre-osteoblast, the said precursors are still able to proliferate, but unable to deposit bone matrix (Neve et al. 2011). Following this, ALP activity will start to increase, and the β-glycerophosphate used in the induction media will act as the source of phosphate ions for the formation of mineralized matrix. Cell proliferation will cease as the osteoblast becomes embedded inside the matrix (Coelho and Fernandes 2000; Franz-Odendaal et al. 2006).

### Molecular characterization of suspension peripheral blood mononucleated stem cell

Molecular characterization was conducted on the suspension cells that had been cultured in medium which can support both the expansion and maintenance of the cell differentiation potential to osteoblast. Thus, based on all the data obtained above, mouse and human suspension PBMNSC cultured in α-MEM, 10 % NBCS and 1 % penicillin–streptomycin were subjected to molecular characterization using RT-PCR after 14 days of expansion in the said medium.

**Fig. 6:**
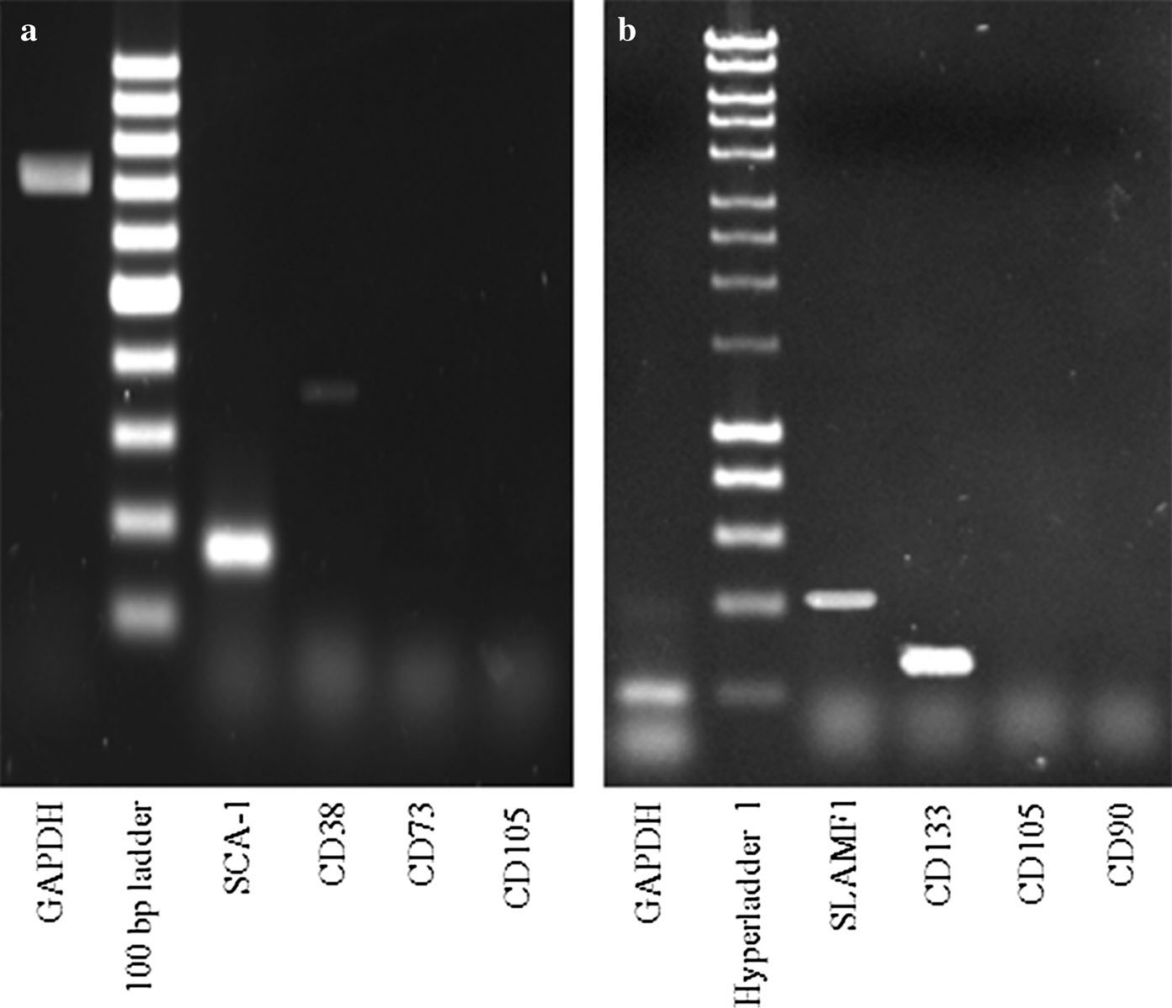
Reverse transcriptase-PCR (RT-PCR) of mice (**a**) and human (**b**) suspension peripheral blood mononucleated stem cells after 14 days cultured in α-MEM + 10 % newborn calf serum (NBCS) + 1 % penicillin–streptomycin. The cells were analyzed for the expression of hematopoietic stem cell marker (mice: SCA-1, CD38; human: SLAMF1, CD133) and mesenchymal stem cell marker (mice: CD73, CD105; human: CD105, CD90). GAPDH was used as internal control for the RT-PCR reaction

Results show that both mouse and human suspension PBMNSC expressed hematopoietic stem cell markers, but not mesenchymal stem cell markers (Fig. 6). These data suggested that the differentiation process of suspension PBMNSC to osteoblast occured through transdifferentiation, signaling the plasticity property of the isolated suspension PBMNSC.

## Conclusion

In this study, it has been proven that both human and mouse suspension peripheral blood mononucleated stem cells (PBMNSC) can be expanded in vitro with just the basal medium and serum without any additional growth factor. The most optimal medium for the proliferation of both mouse and human suspension PBMNSC is determined to be α-MEM supplemented with heat-inactivated newborn calf serum. The medium is able to support self-renewal property of the stem cells and at the same time, maintain their ability to differentiate into osteoblast. The cells cultured in this medium are also positive for hematopoietic stem cell marker and negative for mesenchymal stem cell and osteoblast progenitor markers.
